# Prescribing for the patient and the planet: a pragmatic feasibility study of a behaviour change intervention to reduce liquid amoxicillin prescribing in primary care

**DOI:** 10.1016/j.rcsop.2026.100847

**Published:** 2026-09-18

**Authors:** Adam Pattison Rathbone, Joseph Pickles, Ms Laura Griffiths, Alice McCloskey, Philippa McCabe, Nicola Vasey, Emma Lim

**Affiliations:** a Newcastle University, United Kingdom; b Newcastle upon Tyne Hospitals NHS Foundation Trust, United Kingdom; c Liverpool John Moores University, United Kingdom

**Keywords:** Sustainability, Prescribing, Behaviour change, Infectious diseases, Medication adherence

## Abstract

**Background:**

Oral liquid medicines can be clinically appropriate but may be used when solid oral formulations are feasible, with implications for medicine costs and environmental sustainability. We evaluated the feasibility of GPILL, a theory-informed intervention using prescribing feedback, social norms and information on formulation-associated carbon dioxide equivalent (CO₂e) emissions to support reduced liquid amoxicillin prescribing in primary care.

**Methods:**

The intervention was called ‘GPILL’ which stands for a ‘Green Prescribing Intervention to Lower Liquids’ and was adapted from an existing prescribing feedback intervention based on social normalisation theory which incorporated social-norm feedback. Delivered as an emailed PDF of a letter, the intervention incorporated information about levels of prescribing of the practice in comparison to other practices in the area, the environmental impact of liquid formulations and guidance on supporting patients to swallow capsules rather than use a liquid formulation. A quasi-experimental before-and-after design was used for practices in one geographical area of England. Rates of amoxicillin liquid prescribing were compared to capsules for all practices to generate a ratio of liquid: solid prescribing. Practices in the top 50% received the intervention (*n* = 28); whilst the remaining practices acted as controls (*n* = 26). No patient-level inclusion or exclusion criteria were applied to the practice. Prescribing volumes were obtained from OpenPrescribing, with data extrapolated to calculate costs and carbon dioxide equivalents of a standard course of treatment, using published costs and UK conversion factors.

The primary outcome was to assess the feasibility of the intervention. Secondary outcomes were number of liquid and solid prescriptions, prescribing costs and formulation-associated CO₂e. Outcomes were assessed using routinely collected prescribing data during winter months (December–February) from 2020 to 2024. No deviations from planned outcomes were reported.

**Results:**

It was feasible to identify high liquid-amoxicillin-prescribing practices using routine prescribing data and deliver a low-resource, theory-informed intervention through usual primary-care channels. A greater reduction in liquid prescribing was subsequently observed among intervention practices, providing a preliminary signal that warrants formal effectiveness evaluation. Liquid amoxicillin prescribing decreased by 23% in the intervention practices (from 3549 to 2746 prescriptions; Wilcoxin signed-rank *p* = 0.0287) while increasing by 7% in the control practices (from 2090 to 2234; *p* = 0.175). There was evidence of a statistically significant difference between groups (*p* = 0.01113). Liquid amoxicillin prescribing costs decreased by 50% in intervention practices compared with 29% in controls. Estimated liquid CO₂e emissions decreased by 15% in the intervention group, compared with a 20% increase in controls. This represented an absolute reduction of 131.1 kgCO₂e in the intervention group (z = 2.27, *p* < 0.05) equivalent to charging a smart phone over ten thousand times. These exploratory findings should be interpreted cautiously because allocation was non-random, intervention practices had higher baseline prescribing, and the study was not designed to establish causal effectiveness.

**Conclusions:**

A targeted, theory-informed intervention was feasible and produced a preliminary signal of reduced amoxicillin liquid prescriptions, with associated reductions in costs and estimated formulation-associated CO2e. These findings support the feasibility of a low-cost behaviour change strategy to promote sustainable prescribing in primary care. A larger, prospective study using an appropriate controlled analysis is warranted to determine effectiveness, cost consequences and environmental impact.

## 1 Introduction

Deciding to prescribe an oral liquid dosage form instead of a solid oral dosage form can be complex. For patients who struggle to swallow, or who struggle with coordination, oral liquid formulations may be more appropriate than solid oral dosage forms. However, oral liquid formulations may be overprescribed due to professional or parental misperceptions that children and young people cannot swallow solid dosage forms.^1^, ^2^ The literature here dichotomises the reasons for this broadly into two areas: firstly, the physical characteristics of the medications, such as problems with size, taste, and texture of tablets or capsules.^2^, ^3^, ^4^, ^5^, ^6^ Secondly, the psychological fears about swallowing medications and choking.^7^, ^8^, ^9^

Evidence shows that there are techniques (e.g., KidzMed) to teach children as young as four years old to swallow solid oral dosage forms successfully without choking. Despite these techniques, oral liquid formulations may still be overprescribed, resulting in significant financial costs in some health settings (see below for exact costings).^10^, ^11^ Evidence and clinical practice indicates this may be influenced by parental preferences for oral liquid formulations for young children^12^, ^13^ A qualitative study showed general practitioners and pharmacists considered both the parent's and the child's willingness to use an oral formulation before prescribing a solid oral dosage form in preference to an oral liquid dosage form.^12^ A survey of 1018 school children, adolescents and adult caregivers showed only 32.1% of adult caregivers perceived a solid oral dosage form to be suitable to administer to a child, whilst almost double that proportion (59.6%) of school children reported they would be willing to try it.^13^ This suggests adult caregivers may be hesitant to try a solid oral dosage form, influencing prescribers to prescribe more oral liquid formulations than necessary.

Unfortunately, robust evidence of the prevalence of inappropriate oral liquid medicine prescribing in English primary care is unknown. However, we do know more than 44 million oral liquid medicine items are dispensed annually in primary care, accounting for approximately 4% of all prescription items.^14^ Regional evidence suggests that at least 6% of liquid antibiotic prescriptions may be suitable for substitution with tablets or capsules,^15^ while more than four-fifths of antibiotic prescriptions for children aged 6–12 years have been reported to be liquids.^14^ These findings suggest scope for formulation optimisation, but robust national and international research examining the clinical appropriateness of liquid prescribing is lacking. The authors' own clinical experiences anecdotally suggest that prescribing solid oral dosage forms for some children is not considered as often as it could be due to a normalised culture of prescribing liquids by default for children and young people.

Prescribing behaviours have previously been considered using social normalisation theory.^16^ The theory suggests behaviours and attitudes are normalised within a group or society based on feedback, which in turn leads to an individual behaving similarly to others in the group by conforming, role-modelling, imitating, replicating, or propagating the normalised behaviour. This is important in health services, as prescribers often learn by shadowing others and then go on to work in groups or teams, and so prescribing behaviours may be ‘normalised’. Existing evidence has used social normalisation theory to alter prescribing behaviours in primary care.^17^ Using a database in England for prescribing, a ‘normalised’ level of amoxicillin prescribing was calculated and provided to prescribers, along with their practice-specific level of amoxicillin prescribing. Information about antimicrobial resistance and alternative therapeutic options for prescribers was also provided.^17^ This enabled prescribers to compare their prescribing practices with other prescribers, tapping into the social drivers of behaviours and prompting behaviour change to ‘conform to the norm’.^17^ Providing this information to prescribers led to a statistically significant reduction in the prescribing of amoxicillin.^17^ This evidence demonstrates prescribing behaviours may be changed by providing information about social norms.

However, existing evidence did not focus on a particular dosage form and was dichotomous (prescribe or not to prescribe). Other work has focused on switching inhaler formulation.^18^, ^19^, and demonstrated that switching from oral liquid to solid oral dosage forms can improve adherence, reduce costs for patients, and improve treatment outcomes.^1^ One study showed a month's course of nitrofurantoin liquid was £447 (USD 593.77) compared to tablets, costing only £9 (USD 11.96) for the same course.^1^ The same evidence used a behaviour change technique for children, aimed to support switching from oral liquid to solid oral dosage forms at a public regional children's hospital, which led to a reduction in liquid medicines use and an annual savings of £46,588 (USD 61,885.06).^1^ This complex intervention influenced prescriber behaviour by providing support for them to teach patients to be comfortable swallowing solid oral dosage forms. This suggests there is an opportunity to convert some inappropriate prescription of oral liquid dosage forms into solid oral dosage form, which could improve patient experiences and save money.

More recent evidence suggests switching from liquid to solid oral dosage forms may also reduce carbon emissions. The carbon footprint of liquid amoxicillin is up to threefold higher than a 7-day course of amoxicillin capsules.^20^ Carbon emissions (presented as kilograms equivalent to carbon dioxide or ‘kg CO2e’) for treating Group A streptococcal tonsillitis in a 6-year-old child were 0.093 for 250 mg capsules or 0.066 for 500 mg capsules; compared with 0.307 for 250 mg/5 ml oral suspension.^20^ This is equivalent to the amount of carbon dioxide released when charging twenty-four fully-depleted smartphones.^21^ This is important as there have been recent policy changes in some jurisdictions to change prescribing behaviours to reduce greenhouse gas emissions, for example by identifying global and local influences on disease and adoption of sustainable development goals.^22^, 23., 24.

Although new policy is in itself an intervention to change prescribing behaviours, existing evidence suggests prescribers have a poor understanding of the climate impact of prescribing.^18^, ^25^ Existing behaviour change tools which have included providing information about sustainability were focused on inhalers,^18^, ^19^ rather than switching from oral liquids to solid oral dosage forms. This is problematic as health policies and governments are prioritising a ‘reduced carbon’ approach to health services, but with a limited focus on only inhaled therapies, rather than oral liquid dosage forms, which are much more broadly used. A previous scoping review of literature showed knowledge of sources of carbon dioxide emissions by professionals in primary care was poor.^26^ Behaviour change interventions linking higher levels of oral liquid amoxicillin prescribing with higher greenhouse gas emissions do exist.^27^ However, the feasibility of the intervention was not explored and remains uncertain. Additionally, any intervention would need to be tested to consider the eligible targets, settings, delivery and outcome measures. The gap in the literature, then, is evidence that a behaviour change intervention which uses socially normalised greenhouse gas emissions information to reduce oral liquid formulation prescribing is feasible.

This study, therefore, had a primary objective to pragmatically assess the feasibility of a behaviour change intervention using social-norm feedback to reduce oral liquid formulation prescribing, by assessing changes to i) the absolute number of prescriptions, ii) the cost and iii) carbon dioxide emissions, of solid oral and oral liquid amoxicillin formulations in primary care.

## 2 Methods

We conducted a pragmatic quasi-experimental ecological pre-post feasibility study with a control and intervention group using an approach described by Chan et al.^28^ A pragmatic feasibility study addresses uncertainties relating to an intervention prior to a more robust randomised cluster-controlled trial. Feasibility studies typically set parameters or objectives enabling researchers to identify if delivering an intervention is feasible (or not).^28^ A pragmatic feasibility study considers the effectiveness of an intervention in usual care or practice whilst acknowledging there will be potential measurement errors due to uncertainty and changing outcomes.^28^ Ten domains within a highly pragmatic approach allow researchers to identify areas of uncertainty when conducting feasibility studies. These domains can be used to assess the feasibility of the intervention as met, partially met or not met. The details of the intervention, data collection and analysis are set out below using the TiDIerR Checklist to support transparency (see supplemental materials).^29^

### 2.1 Patient and Public Involvement

It was not possible to involve patients or the public in the design, conduct, reporting or dissemination plans for our research. Patients and the public were not involved in this feasibility study because resources to support their engagement were not available. However, patient and public involvement will inform the design of the subsequent definitive study.

### 2.2 Intervention

The development of the green prescribing intervention to lower liquid (GPILL) medicines use is described in previously published work.^27^ The intervention was based on previous work seeking to reduce antimicrobial use,^17^ which was adapted using the Theoretical Domains Framework and Capability, Opportunity, Motivation – Behaviour model.^30^, ^31^, ^32^, ^33^ This model posits that behaviours are enacted if individuals have the physical and mental capability, the external or intrinsic motivation, as well as the social and environmental opportunity to perform the behaviour. In this context, the intervention aimed to provide prescribers with the *capability* to support patients to switch from an oral liquid to a solid oral dosage form by signposting to resources; an *opportunity* to change behaviour by highlighting their current levels of liquid and solid oral dosage form prescribing and *motivation* for themselves and patients using normalised feedback on greenhouse gas emissions. For further information about the development of the intervention, please see Pickles, Griffiths, McCloskey, Vasey, Lim, Rathbone.^27^

The intervention creates motivation primarily based on the principle of social-norm feedback.^16^ This theory describes behaviour as an individual's conformity to idealised standards or accepted behavioural norms within a group.^16^ This theory was chosen as it has previously been used successfully to reduce amoxicillin prescribing in primary care by providing ‘normal’ levels of amoxicillin prescribing alongside practice-specific levels of amoxicillin prescribing.^17^ The intervention was adapted to include additional information about sustainability, establishing a ‘social norm’ of greenhouse gas emissions using data extracted from a publicly available database of raw prescribing data in England.^34^, ^35^, 36. The dataset relates to the number of prescriptions for individual formulations listed in the British National Formulary, for each practice and pools data from the Office for National Statistics, NHS Business Services Authority and NHS Digital.^36^ Access to the database is via the OpenPrescribing.net platform, administered by the Bennett Institute for Applied Data Science at the University of Oxford, which enables users to conduct searches across specific practices, organisations and regions for specific drug formulations or drug classes, using the classification system from the British National Formulary.^36^

The intervention was constructed as a letter (see supplemental material), addressed to prescribers, which included information about the level of amoxicillin liquid prescribing for the practice, the average level of amoxicillin liquid prescribing for other practices in the region, and the estimated difference in carbon emission equivalences. The intervention also included information about supporting patients to switch from oral liquid to solid oral dosage forms and signposted to further areas of support (see Supplementary Information). The intervention was reviewed by primary care prescribing stakeholders, including physicians, pharmacists, practice managers, and prescribing clerks, and adapted based on their feedback.^27^ Adaptations included the volume of information, editing to keep the information on a single A4 page, and who the letter should be sent from (someone with appropriate social and clinical influence, such as a prescribing lead for the area). Practices were invited to contact the team for further information or training, and although training was offered, practices did not take this up. Intervention adherence was assessed by three authors (LG, JP, APR) who kept a log of when the intervention was sent and monitored read receipts. The intervention was sent as specified to all 28 intervention practices; read receipts confirmed opening by 19 practices.

The intervention was sent once to practices identified in the top 50% of prescribers of oral liquid amoxicillin in comparison to solid oral formulations based on a Liquid: Solid Prescribing Ratio in one region of the health service in England. This was calculated using data from OpenPrescribing (see Table 3) and the following formula:

$$=\left(\;\frac{\mathrm{number of liquid amoxicillin prescriptions}}{\mathrm{number of}\;\mathrm{all}\;\mathrm{antimicrobial prescriptions}}\;\right)÷\left(\;\frac{\mathrm{number of solid amoxicillin prescriptions}}{\mathrm{number of}\;\mathrm{all}\;\mathrm{antimicrobial prescripti}o\mathrm{ns}}\right)$$

The ratio enables consideration to be made for differences between practices. Data was extracted from OpenPrescribing for 59 practices in one geographical region and manipulated in Microsoft Excel by one author (APR) and checked by two authors (LG, JP). Practices were ranked from highest to lowest, and the practices in the highest 50% of Liquid: Solid Prescribing Ratio (*n* = 28) were sent the intervention in December 2023 by two authors (LG, JP) via a single email to publicly available email addresses (read receipts received *n* = 19).

### 2.3 Data collection

Searches were conducted using OpenPrescribing.net‘s interface using search terms relating to all available oral liquid and solid oral preparations of amoxicillin (see Table 3) in the ‘Search GP prescribing data’ function. The denominator for the searches (shown in the ‘versus' option, drugs or BNF sections) was ‘Antibacterial drugs (5.1)’. The ‘highlighting’ option was set to ‘practices' for ‘NHS NEWCASTLE GATESHEAD (13T)’. Data were downloaded as CSV files and prepared for analysis using Microsoft Excel. From each raw data set, data relating i) number of oral liquid or solid items prescribed (y items), ii) cost of liquid items prescribed (y actual cost), iii) number of antibacterials prescribed (x items) and iv) cost of antibacterials prescribed (x actual cost) for the study period, for each practice, for each month of the study period were extracted and merged into a single spreadsheet. Data were collected in April 2025 for the following study periods Winter 2020 relates to data from December 2020 – February 2021, Winter 2021 relates to data from December 2021 – February 2022; Winter 2022 relates to data December 2022 – February 2023 (immediately pre-intervention) and Winter 2023 relates to data from December 2023 to February 2024 (post-intervention). Data were collected for multiple years pre-intervention to show changes in prescribing behaviours such as stock supply issues, Group A Streptococcal outbreak, and changing costs. This enabled ‘trajectories' of prescribing to be made and considered when interpreting the results.

### 2.4 Data analysis

Descriptive statistics were calculated using functions in Microsoft Excel. Differences between the number of prescriptions, cost of prescriptions, and carbon dioxide emissions were calculated by subtracting data collected after the intervention from data collected before the intervention. Calculations to estimate carbon dioxide equivalent totals per practice, for the study period, per year, assumed each prescription related to either i) amoxicillin 250 mg capsules TDS for 7 days for solids or ii) amoxicillin 250 mg/5 ml TDS for 7 days for liquids and used published carbon dioxide equivalents of 0.093 kg CO2e for capsules and 0.307 kg CO2e for liquid.^20^ Only data relating to amoxicillin 250 mg capsules and amoxicillin 250 mg/5 ml oral solution and oral sugar-free solution were used in the carbon dioxide equivalent calculations. Data searches and descriptive statistical analysis were carried out by one author (APR) and verified by a second author (APM). Inferential statistical analysis (Wilcoxon-Signed Rank Test, Mann-Whitney *U* Test) was carried out by one author (APR) using Microsoft Excel (Version 2502), checked by a second author (PMc) using RStudio^37^ (Version 2024.12.1 + 563) and verified by all other authors. Significance is reported at the alpha value of 0.05.

The primary purpose of the study was to assess the feasibility of implementing and evaluating the GPILL intervention using routinely collected prescribing data. Feasibility was assessed using domains adapted from Chan et al., including eligibility, recruitment, setting, delivery, adherence, follow-up and outcome assessment. Statistical analyses of prescribing outcomes were exploratory and were not intended to provide definitive estimates of intervention effectiveness. The Shapiro-Wilk Test showed data were not normally distributed, and so non-parametric tests were conducted. The Wilcoxon Signed-Rank (WSR) test was performed on the number of oral liquid prescriptions in the intervention and control groups to assess if differences pre- and post-intervention were significant for the absolute number of prescriptions. Statistical significance was not assessed for differences in absolute costs, liquid-to-solid prescribing ratio.

### 2.5 Ethics

Ethical approval for the study was obtained from Newcastle University Faculty of Medical Sciences Research Ethics Committee (36,416/2023). The Health Research Authority's ‘Is my study research?’ tool was used (available https://www.hra-decisiontools.org.uk/research/) and identified that this study did not require further system-level ethical approval.

## 3 Results

### 3.1 Feasibility

Feasibility was assessed using the domains outlined by Chan et al.^28^ (see Table 1). Based on the feasibility findings, the GPILL intervention appears practical to deliver in routine primary care, requiring minimal additional resources and using existing prescribing data and communication routes. Most feasibility objectives were met, including recruitment, eligibility, setting, delivery, adherence, follow-up and relevance of the primary outcome, although some additional expertise may be needed to generate and communicate carbon-emissions data for wider implementation. These findings support progression to a larger, appropriately powered trial to confirm effectiveness, explore the mechanisms driving behaviour change, and assess whether the intervention can be implemented consistently across different primary care settings

**Table 1:** Feasibility objectives adapted from Chan et al.^28^ domains of uncertainty for a pragmatic feasibility study.

PRECIS-2 Domain	Feasibility objectives	Application to this study	Feasibility objective
Intervention development	If effective, the intervention would be ready and acceptable for implementation in usual care	The behaviour change intervention is low cost and can be implemented with minimal additional resources	Met
Research ethics	Adopt waived forms of consent to minimize additional burden over usual care procedures	The behaviour change intervention did not require ethical approval at a system level however institutional approval was granted	Met
Eligibility	Include participants in the study that are similar to those who would receive the intervention if it were part of usual care	Practices were included in the study who had high levels of liquid amoxicillin prescribing in comparison to solid amoxicillin prescribing.	Met
Recruitment	Recruit participants with no more effort than would be used in usual care to engage with patients	Practices were identified simply using routinely collected, publicly available data	Met
Setting	Include a range of centres and settings similar to where the results are intended to apply	Practices were based in a range of different socioeconomic settings in one region.	Met
Organization	Use no more resources, provider expertise, or organizational structure than those available in usual practice	Minimal resources were required and organizational structures already existed. However, providers would not routinely be aware of the carbon emissions linked to prescribing of liquid medicines. This expertise was available to authors due to previous research work and is not routinely available in relation to other liquid medications so would require additional resources or expertise in comparison to usual practice for further trials	Met – partially
Flexibility of delivery	Deliver the intervention with the same flexibility that is anticipated in usual care, often leaving the details of how to implement the intervention up to the providers	The behaviour change intervention was emailed to practices, with uncertainty and flexibility with how the intervention was processed or disseminated once received. This reflects usual communication processes in primary care practice.	Met
Flexibility of adherence	Allow participants to engage with the intervention with the same variability that is anticipated in usual care, monitoring and encouraging adherence no more than would take place in usual care	Adherence to the intervention was not controlled in this study but was monitored only with read receipts and so reflected variability seen in usual practice.	Met
Follow-up	Data collection and follow-up guided by usual care practices	The number of prescriptions of solid oral and oral liquid amoxicillin is routinely collected and guided by usual practice.	Met
Primary outcome	Selects a primary outcome that is directly relevant	The primary outcome was the number of prescriptions for oral liquid and solid oral amoxicillin, which was directly relevant to the intervention	Met

The intervention was sent to 28 practices in the intervention group and 26 in the control group (see Table 2) Data were removed relating to five practices due to incompleteness in pre- or post-intervention data. Practices in the intervention group had a higher level of oral liquid amoxicillin prescribing compared to solid oral amoxicillin prescribing in the year before the intervention (Liquid: Solid Prescribing Ratio of 0.40 versus 0.25). Oral liquid amoxicillin in the intervention group prior to the intervention equated to approximately £18,909.28 (USD 25,156.02) compared to £11,518.60 (USD 15,323.81) in the control group. Practices in the control and intervention groups were not matched according to size, patient cohort demographics or any other practice-specific information. Only information relating to the number and cost of solid oral and oral liquid forms of amoxicillin was used for the study, with practices in the top 50% of prescribers being recruited into the intervention group.

**Table 2:** Summary of secondary outcomes following the GPILL intervention.

Outcome	Intervention practices		Control practices		Interpretation and analysis
	Before	After (% change)	Before	After (% change)	
Oral liquid amoxicillin prescriptions (n)	3549	2746 (**−23%)**	2090	2234 (+7%)	Wilcoxon signed-rank: intervention *p* = 0.0287; control *p* = 0.175. Liquid prescribing significantly decreased in the intervention group whilst no significant change occurred in control practices
Liquid amoxicillin prescribing ratio	0.40	0.38 (**−5%)**	0.25	0.31 (+24%)	The relative use of liquid formulations decreased in intervention practices but increased in controls, representing an approximate 29 percentage-point difference in change between groups.
Cost of oral liquid amoxicillin (£)	£18,909	£9403 (**−50%)**	£11,518	£8170 (**−29%)**	Costs decreased in both groups, but the reduction was 21 percentage points greater in intervention practices. This is equivalent to -£9505.63 in intervention practices and -£3348.36 in control practices
Estimated oral liquid amoxicillin emissions (kgCo2e)	857.14	726.06 (**−15%)**	508.09	611.24 (+20%)	Mann–Whitney: z = 2.27, *p* < 0.05. Estimated emissions decreased in the intervention group but increased in controls, with a statistically significant between-group difference. In the intervention group this was equivalent to a reduction of −131.09kgCO_2_e and an increase in the control group of +103.15kgCO_2_e – an overall difference of 233.24kgCO_2_e

Notes: Wilcoxon signed-rank tests examined pre–post changes within groups. The Mann–Whitney *U* test compared intervention and control practices. Cost and liquid ratio outcomes were analysed descriptively. Estimated formulation-associated CO2e were estimated using standardised published kgCO₂e values rather than directly measured emissions.

**Table 3:** Open prescribing search terms.

Name	Code	Versus	Highlighting
Liquids		Drugs or BNF sections - ‘Antibacterial drugs (5.1)’	Practices – ‘NHS NEWCASTLE GATESHEAD’
Amoxicillin 125 mg/1.25 ml oral suspension paediatric	0501013B0AAAIAI		
Amoxicillin 125 mg/5 ml oral suspension	0501013B0AAAJAJ		
Amoxicillin 125 mg/ml oral suspension sugar-free	0501013B0AAASAS		
Amoxicillin 250 mg/5 ml oral suspension	0501013B0AAAKAK)		
Amoxicillin 250 mg/5 ml oral suspension sugar-free	0501013B0AAATAT		
Amoxicillin 500 mg/5 ml oral suspension sugar-free	0501013B0AAAVAV		
Solids			
Amoxicillin 250 mg capsules	0501013B0AAAAAA		
Amoxicillin 500 mg capsules	0501013B0AAABAB		
Amoxicillin 500 mg tablets	0501013B0AABDBD

**Table 4:** Absolute number of solid oral amoxicillin, liquid oral amoxicillin, and antimicrobials prescribed in winter months (December – February) and percentage difference to the previous year

	Number of prescriptions				% difference to the previous year		
	2020	2021	2022	2023⁎	2021	2022	2023⁎
Number of Solid Oral Amoxicillin Prescriptions							
Intervention practices	3459	5509	8992	7313	59%	63%	−19%
Control practices	3707	5851	8562	7311	58%	46%	−15%

Number of Oral Liquid Amoxicillin Prescriptions							
Intervention practices	651	1626	3549	2746	150%	118%	−23%
Control practices	437	1165	2090	2234	167%	79%	7%

Number of Antimicrobial prescriptions							
Intervention practices	27,321	32,008	42,617	39,260	17%	33%	−8%
Control practices	27,710	32,537	39,327	37,528	17%	21%	−5%

Data extracted from OpenPrescribing.net Bennett Institute for Applied Data Science, University of Oxford, 2025.

⁎ denotes the year a behaviour change intervention informing prescribers of the greenhouse gas emissions linked to liquid amoxicillin use was sent to practices in the intervention group.

**Table 5:** Total cost of solid oral amoxicillin, oral liquid amoxicillin, and all antimicrobials prescribed in one region.

	Estimated costs of prescriptions				% differences to the previous year		
	2020	2021	2022	2023*	2021	2022	2023*
Solid oral amoxicillin prescription costs							
Target practices	£ 15,923.38	£ 24,022.63	£ 32,996.92	£ 9562.80	51%	37%	−71%
Control practices	£ 5390.99	£ 8062.78	£ 30,736.25	£ 9494.23	50%	281%	−69%

Oral liquid amoxicillin prescription costs							
Target practices	£ 3641.32	£ 4338.90	£ 18,909.28	£ 9403.65	19%	336%	−50%
Control practices	£ 1142.75	£ 2133.59	£ 11,518.60	£ 8170.24	87%	440%	−29%

Antimicrobial prescription costs							
Intervention practices	£ 615,743.26	£ 583,662.81	£ 291,712.57	£ 223,051.86	−5%	−50%	−24%
Control practices	£ 209,999.74	£ 200,339.33	£ 307,063.85	£ 247,732.68	−5%	53%	−19%

Shows estimated costs of prescriptions between December and February (winter months) per year. Data extracted from OpenPrescribing.net, Bennet Institute for Applied Data Science, University of Oxford, 2025. * denotes the year a behaviour change intervention informing prescribers of the greenhouse gas emissions linked to liquid amoxicillin use was sent to practices in the intervention group.

**Table 6:** Total greenhouse gas emissions (kgCO2e) for solid and liquid amoxicillin preparation over time.*

	Winter Period				% difference to the previous year		
	2020	2021	2022	2023⁎	2021	2202	2023⁎⁎
Total Solid kgCO2e							
Intervention	6.0450	6.4170	11.7180	7.5330	6%	83%	−36%
Control	6.8820	7.8120	13.7640	10.6950	14%	76%	−22%

Total Liquid kgCO2e							
Intervention	153.5000	399.1000	857.1440	726.0550	160%	115%	−15%
Control	113.2830	297.7900	508.0850	611.2370	163%	71%	20%

Assumes all solid prescriptions in the study period were for amoxicillin 250 mg Capsules TDS for 7 days, and all liquid prescriptions were for amoxicillin 250 mg/5 ml oral solution or oral sugar-free solutions TDS for 7 days. Data taken from OpenPrescribing.com, The Bennet Institute of Applied Science, Oxford University. Calculation used assumes typical kgCO2e for amoxicillin 250 mg Capsules TDS for 7 days was 0.093 kgCO2e, and amoxicillin 250 mg/5 ml oral solution or oral sugar-free solutions TDS for 7 days was 0.307 kgCO2e from Parker et al. (2023) EcoKidzMed: Measuring the carbon emissions of packaging, distribution, and waste disposal of liquid and capsule amoxicillin. BMJ Publishing Group Ltd.

⁎ Denotes the year a behaviour change intervention informing prescribers of the greenhouse gas emissions linked to liquid amoxicillin use was sent to practices in the intervention group.

### 3.2 Absolute number of prescriptions

The findings show an absolute reduction in the total number of oral liquid amoxicillin prescriptions in the intervention group compared to the previous year (*n* = 3549 vs 2746), equating to a 23% reduction (see Table 4). Practices in the control group showed a 7% increase in the absolute number of oral liquid amoxicillin prescriptions compared to the previous year (*n* = 2090 vs 2234). The WSR test indicates changes in the absolute number of prescriptions pre- and post-intervention were statistically significant in the intervention group (*p* = 0.0287), however, not in the control group (*p* = 0.175).

A Mann–Whitney *U* test was used to compare the number of oral liquid amoxicillin prescriptions between intervention and comparison practices during Winter 2023. Median prescribing was 32.0 prescriptions [IQR 21.0–41.0] in intervention practices and 24.0 [14.0–35.75] in comparison practices (*p* = 0.01113, *r* = 0.199). Practices were allocated according to baseline prescribing rather than randomised, this between-group comparison should not be interpreted as an estimate of intervention effectiveness.

Overall, both practices in the intervention and control group prescribed less oral solid amoxicillin compared to the previous year (*n* = 8992 vs 7313 and *n* = 8532 vs 7311), with practices in the intervention group showing a larger percentage reduction compared to practices in the control group (−19% vs −15%, respectively). Similarly, overall antimicrobial prescribing was also reduced for practices in both the intervention and control group compared to the previous years (*n =* 42,617 vs 39,260 and *n* = 39,327 vs 37,528, respectively), again with practices in the intervention group showing a larger reduction compared to practices in the control group (−8% vs −5%, respectively).

### 3.3 Liquid: Solid prescribing ratio

As a ratio, the number of oral liquids to solid oral amoxicillin prescribing showed a decrease in practices in the intervention group but was increased in the control group (*n =* 0.4 vs 0.38 and 0.25 vs 0.31). This indicates a reduction of approximately 5% in comparative prescribing in the intervention group, compared to an increase of approximately 24% in the control group – a total of 29% difference in relative change between groups.

### 3.4 Costs

Absolute costs of oral liquid amoxicillin prescriptions were reduced in both intervention and control groups compared to the previous year, with a larger reduction for practices in the intervention group compared to the control group (from £18,909.28 to £9403.65 in the intervention group and from £11,518.60 to £8170.24 in the control group). This indicates a 50% and 29% reduction, respectively (see Table 5). These findings were also echoed in the costs of prescriptions. This suggests the relative reduction in costs of approximately 21% between intervention and control practices.

Costs of solid oral amoxicillin prescriptions and all antimicrobial prescriptions were also reduced compared to the previous year in both intervention and control groups, though reductions in costs were smaller than for oral liquid amoxicillin prescriptions. Costs were reduced by 2% for solid oral amoxicillin, by 5% for all antimicrobials, and by 21% for oral liquid amoxicillin prescriptions.

### 3.5 Carbon reduction

Practices in the intervention group had an estimated reduction of 15% in greenhouse gas emissions compared to an increase of 20% in the control group, compared to the previous year (See Table 6). On average, amoxicillin 250 mg/5 ml greenhouse gas emissions were 8.6435 kg CO2e in the intervention group and 7.8364 kg CO2e in the control group. Data were non-normally distributed, and the Mann-Whitney Test for two independent samples shows a z-score of 2.27; this exceeds 1.96, *p* < 0.05, suggesting a statistically significant difference between the greenhouse gas emissions of practices in the intervention group compared to the control group. The total greenhouse gas emissions for liquid amoxicillin in the intervention group were 857.14 kgCO2e and 726.05 kgCO2e in the intervention group. This is a difference of 131.0890 kgCO2e. In the control group, total greenhouse gas emissions for liquid amoxicillin increased from 508.08 to 611.23 kgCO2e, an increase of 103.15 kgCO2e.

## 4 Discussion

The findings suggest providing social-norm feedback on liquid prescribing and estimated CO₂e emissions is a feasible approach to reducing liquid amoxicillin prescribing, associated costs and estimated emissions. This is important because the project findings are extending prior behaviour change work by applying social norm feedback to sustainability-oriented prescribing outcomes. However, as a pragmatic feasibility study there is uncertainty and error attributed to the dated collected which means further, appropriately powered work, needs to be conducted to identify the mechanism of the intervention and demonstrate efficacy.

### 4.1 Implications for research

This work builds on existing evidence that behaviour change interventions can alter prescribing practices in primary care.^17^ There is limited literature which describes comparative behaviour change interventions, with existing work targeting the prescription of anaesthesia^25^ or inhalers^19^ and using a range of intervention types, such as changes to national guidelines or payment systems,^19^ as well as multi-faceted interventions (such as toolkits), patient-mediated, educational, and audit or feedback interventions.^38^ Reducing inhaler prescribing in relation to greenhouse gas emissions was described by Franklin, Mallen, Twohig.^18^ In their work, health professionals in primary care were asked about their attitudes to reducing pressurised metered dose inhalers and prescribe dry powder inhalers (DPIs) instead. Their findings showed prescribing decisions were driven by patient-specific factors, such as the ability of the patient to use the formulation correctly; social factors, such as family experiences of using the formulation, and biases and assumptions about the patients themselves (e.g. participants were less likely to prescribe DPIs for older patient's due to assumptions about dexterity.)^18^ The confidence and knowledge of clinicians as well as how their practice fitted in to the status quo of asthma care was also reported to influence prescribing behaviours.^18^ In contrast, participants feared stigma if they discussed climate considerations of prescribing, with tension between workload and time pressures to do the right thing for the patient and addressing climate concerns to do the right thing for the planet.^18^ The authors summarised their findings, stating the barriers to changing prescribing behaviours to reduce greenhouse gas emissions included a lack of understanding about carbon footprint, ingrained cultural assumptions, clinical inertia and poor understanding of formulation suitability for certain groups.^18^ Although their work used qualitative methods and did not assess effectiveness, it provides context to the findings presented in our study. The barriers to changes to prescribing behaviour identified by Franklin, Mallen, Twohig^18^ appear to be addressed (in some part) by the behaviour change intervention used in this study by providing clinicians with information about different formulations alongside how those formulations can be used safely in specific groups (such as children) and how greenhouse gas emissions can be understood by using estimated equivalences. This work then goes beyond existing literature by demonstrating the feasibility of modifying prescribing behaviours using interventions which include socially normalised information about greenhouse gas emissions alongside alternative therapeutic options.

Existing work has identified that reducing inappropriate prescribing in primary care are key targets for reducing carbon emissions in the real world.^19^, ^26^, ^39^, 40. A study which compared changes to prescribing behaviour across the UK showed pronounced changes to Welsh prescribing of inhalers using a complex intervention which included the publication of a toolkit with updated guidance about the carbon footprint of different inhalers, alongside webinars, video-based educational updates and a digital app aimed at both patients and professionals.^19^ That work used a large sample and was able to identify the effectiveness of interventions rolled out at different times across the different UK nations; showing definitively that changes to prescribing behaviours were more pronounced following the publication of the Welsh toolkit in comparison to other interventions rolled out in England, Northern Ireland and Scotland, for example, which included incentivised payments which had little effect.^19^

A systematic review of interventions to change prescribing behaviour shows the majority are effective, whilst education-based interventions are less effective than other types of interventions such as multi-faceted, patient-mediated and audit or feedback interventions.^38^ This is likely influenced by publication bias, where only positive interventions are published, and so should be interpreted with some caution. That our intervention was feasible despite being much simpler and straight forward to deliver than a complex intervention is important and suggests smaller interventions may also be feasible in the fight against climate change. However, the study provides preliminary signal of effectiveness in routine practice but was not designed or powered to establish efficacy, including how reliable the intervention is at changing behaviour over time in different groups or how effective it is. Further work is therefore needed to identify the efficacy of the intervention, in an appropriately powered comparative trial.

Other work is currently using a factorial randomised online hypothetical experiment to explore changes to prescribing behaviour by providing different types of environmental impact information to prescribers, though this is in the context of inhalers and has yet to report results.^41^ Where as in our study information was presented alongside socially normalised estimates but other work could include drawing equivalence between the reduced carbon dioxide emission and other aspects of everyday life.^21^ This could include explaining to prescribers that a reduction of 131 kgCO2e in total liquid amoxicillin prescribing for a three-month winter period equates to roughly driving 334 miles by an average gasoline-powered car, burning 146 pounds of coal, or charging a fully-depleted smartphone 10,598 times. Helping practitioners understand the volumes of carbon equivalencies in ‘real’ terms may enable them to balance the tension between prescribing for the patient and the planet. Although helpful, future work should agree on standard approaches to presenting carbon equivalences, as at the moment these can vary across jurisdictions with disputed validity between conversion factors.^42^

Although there are still many uncertainties about the mechanism of the intervention in providing socially normalised greenhouse gas emissions linked to prescribing, this study does provide promising preliminary evidence that changes to existing interventions, such as the one originally developed to reduce antimicrobial resistance,^17^ may be repurposed or refocused to combat modern challenges, such as climate change. Another key point of learning for researchers here then is not to ‘re-invent the wheel’ but to ‘re-cycle and re-use’ methods – a key part of reducing carbon emissions in health services and social pharmacy research.

### 4.2 Strengths and limitations of this study

The strengths of the project lie in it's pragmatic design and use of routinely collected prescribing data in primary care. This objective, routinely collected OpenPrescribing data reduced participant and researcher burden and enabled assessment of clinically and policy-relevant outcomes including prescribing volumes, cost and estimated formulation-associated CO2e. The intervention itself was simple and low cost, which made it easy to deliver in the real-world. The intervention was developed using an established behaviour change theory which provides a strong conceptual basis for how and why prescribing behaviour might change.

The research includes limitations due to the pragmatic methodological design, which makes it difficult to know if the effect was due to the intervention or a confounding variable. For example, practices were not matched for specific characteristics (e.g. list size, patient cohort demographics, employee demographics, practice organization). Additionally, the practices may have been exposed to extraneous variables which have yet to be identified, which occurred pre- and post-intervention, e.g. other campaigns to reduce greenhouse gas emissions from prescribing. This is important as there was a background reduction in overall antimicrobial prescribing, suggesting prescribing may have also been influenced by other factors, such as the publication of toolkits such as the 10.13039/501100003924Royal Pharmaceutical Society's Greener Pharmacy Toolkit,^43^ which provides structure and support for practitioners to integrate carbon reduction principles into their practices. The intervention group also had higher oral liquid prescribing levels before the intervention, which could falsely make the intervention effect appear larger, sometimes called ‘regression to the mean’.^44^ This has been shown in other work using data sets evaluating prescribing in primary care.^44^ The design also may be subject to the Hawthorne Effect, whereby behaviours of research subjects are altered simply because they are being evaluated, and not due to the intervention under evaluation.^45^ This means the findings may overestimate the effect of the intervention. Additionally, the data set is collected from reimbursement claims from dispensing contractors, so it may not include prescriptions that were written but never dispensed which may underestimate the levels of prescribing. From a statistical perspective, non-parametric tests are less informative than parametric tests.^46^ However, non-parametric tests are considered to be useful for small studies, with non-normal distributions, like this one.^47^

Additionally, there are weaknesses implicit in the data and method used to estimate emissions as this draws on standardised estimates, which introduces errors which are not accounted for. This is important as this study did not actually measure carbon content of the atmosphere, but instead inferred findings based on estimates. Although technical, the compounding effect of error is important to accurately describe what has been done and what has been learned from this study. It is therefore critical that further work is conducted, which is appropriately powered, accounts for errors and transparently reports findings.

Further work should look to use multiple sources of prescribing and dispensing data, for example, from individual practices as well as pharmacy claims data, to triangulate the impact of the intervention. The term ‘solid oral dosage form’ was used in this study to refer to amoxicillin capsules; however, in the literature and practice, this term is used to describe tablets, capsules, pills, and some granules. In the English setting of this study, the data related largely to amoxicillin capsules (though data for amoxicillin tablets was also included in the searches on OpenPrescribing), and so caution must be used when applying the findings to other jurisdictions where other types of amoxicillin solid oral or oral liquid dosage forms are used, which may have less significant differences in costs and carbon dioxide equivalent emissions (e.g. 50 ml amoxicillin liquid).

## 5 Conclusion

The findings suggest it is feasible to provide social norm feedback about greenhouse gas emissions to high prescribers of oral liquid amoxicillin in comparison to solid oral amoxicillin, alongside information about teaching patients to swallow tablets without choking and signposting to further areas of support. We observed a reduction in the absolute number of amoxicillin liquid prescriptions, reducing associated costs and formulation-associated CO_2_e where practices received the intervention which were not observed in practices which did not receive the intervention. The non-randomised design and baseline imbalance prevent concluding causal attribution and so a larger prospectively designed comparative study is required to determine effectiveness.

## Data statement

Data is available upon reasonable request to the corresponding author via email.

## CRediT authorship contribution statement

**Adam Pattison Rathbone:** Writing – review & editing, Writing – original draft, Visualization, Validation, Supervision, Software, Resources, Project administration, Methodology, Investigation, Funding acquisition, Formal analysis, Data curation, Conceptualization. **Joseph Pickles:** Writing – review & editing, Writing – original draft, Visualization, Validation, Software, Project administration, Methodology, Investigation, Formal analysis, Data curation. **Ms Laura Griffiths:** Writing – review & editing, Writing – original draft, Visualization, Validation, Supervision, Software, Resources, Project administration, Methodology, Investigation, Funding acquisition, Formal analysis, Data curation, Conceptualization. **Alice McCloskey:** Writing – review & editing, Writing – original draft, Visualization, Validation, Supervision, Software, Resources, Project administration, Methodology, Investigation, Funding acquisition, Formal analysis, Data curation, Conceptualization. **Philippa McCabe:** Writing – review & editing, Writing – original draft, Visualization, Validation, Supervision, Software, Resources, Project administration, Methodology, Investigation, Funding acquisition, Formal analysis, Data curation, Conceptualization. **Nicola Vasey:** Writing – review & editing, Writing – original draft, Visualization, Validation, Supervision, Software, Resources, Project administration, Methodology, Investigation, Funding acquisition, Formal analysis, Data curation, Conceptualization. **Emma Lim:** Writing – review & editing, Writing – original draft, Visualization, Validation, Supervision, Software, Resources, Project administration, Methodology, Investigation, Funding acquisition, Formal analysis, Data curation, Conceptualization.

## Funding

No funding to be declared.

## Declaration of competing interest

During the preparation of this work, the authors used ChatGPT in order to create an initial draft. After using this tool/service, the authors reviewed and edited the content as needed and take full responsibility for the content of the published article.

The authors declare that they have no known competing financial interests or personal relationships that could have appeared to influence the work reported in this paper.
