# Supplementary material for: Prescribing for the patient and the planet: a pragmatic feasibility study of a behaviour change intervention to reduce liquid amoxicillin prescribing in primary care
Source: Explor Res Clin Soc Pharm. 2026 Sep 18;24:100847. doi: 10.1016/j.rcsop.2026.100847 (PMC13632174; doi:10.1016/j.rcsop.2026.100847)
Supplement: Supplementary file 1 [file mmc1.docx]

[Gp Name][Address 1, Address 2] [Address 3, Address 4]

[Date]

**NOTE TO PRACTICE MANAGER PLEASE FORWARD IMMEDIATELY TO PRESCRIBING LEAD**

Dear Dr [GP_surname]

**We identified your practice is a high prescriber of liquid Amoxicillin formulations.**

We know that last year, many pharmacies ran out of liquid Amoxicillin during the Group A Streptococcus (GAS) outbreak. We understand there has been an increase in GAS infections in Australia in the last six months and so we can predict this may occur here in the UK, this winter. In anticipation, we would encourage you to support patients to practise swallowing pills in case another shortage occurs.

Our research showed **carbon emissions** **were three times** **lower for a 7-day course Amoxicillin pills compared to** **liquids**. Carbon emissions (in kg CO2e) for treating group A streptococcal tonsillitis in a 6-year-old child were 0.093 for 250mg capsules or 0.066 for 500mg capsules and 0.307 for 250mg/5ml oral suspension. Antimicrobial resistance and climate change are serious threats to our health. Reducing unnecessary prescriptions of liquid antibiotics may help prevent a climate catastrophe. **Last year, Amoxicillin liquid prescribing at your practice equates to the equivalent of [insert value] carbon dioxide.**

KIDZMED is an educational package to help children and families learn to how to swallow pills from the age of 4 years old. Our experience in hospital has shown **children prefer pills to liquids as they taste better, but they also last longer, don’t need to be stored in the fridge and are substantially cheaper** - the Great North Children’s Hospital has saved more than £50,000 per year from switching children from liquids to pills.

**Your practice prescribes more liquid formulations of Amoxicillin compared to pills than other practices in your area.***

Many practices are already taking action to reduce liquid antibiotic prescriptions whilst safeguarding patient’s health. Please join them by taking three simple actions:

1. Give any patients over 4 years old or with pill aversion **advice on how to swallow pills** – you can search online for “KidzMed” [Swallowing pills (Kidzmed) :: North East and North Cumbria Healthier Together (nenc-healthiertogether.nhs.uk)](https://www.nenc-healthiertogether.nhs.uk/parentscarers/medicine-children/pill-swallowing-kidzmed) or visit <https://tinyurl.com/pillschool>
2. Consider **offering a back-up prescription of liquid** if the patient is unable to complete a course of tablets or capsules – this could be post-dated or collected by the patient after trying a course of tablets or capsules if necessary.
3. **Talk to other prescribers** in your practice to ensure that they are also acting to reduce prescribing liquid formulations – data on prescribing is recorded at practice level.

We know that prescribers are aware of the problem and that prescribing is not a simple issue. There are small changes we can all make that will have a big effect on everyone’s health.

Please help us combat climate change from medicines.

Yours faithfully,

**Dr Emma Lim**

Consultant Paediatrician Infectious Diseases and General Paediatrics.

**Mrs Nicola Vasey**

Senior Lead Clinical Pharmacist for Women’s and Children’s Services

**Dr Michael McKean**

Clinical Advisor for Policy, North East and North Cumbria Child Health and Wellbeing Network, Vice-President for Policy, Royal College of Paediatrics and Child Health
